# Self-reported receipt of healthcare professional’s weight management counselling is associated with self-reported weight management behaviours of type 2 diabetes mellitus patients

**DOI:** 10.1186/s40064-016-2029-4

**Published:** 2016-03-29

**Authors:** Victor Mogre, Peter Wanaba, Peter Apala, Jonas A. Nsoh

**Affiliations:** Department of Human Biology, School of Medicine and Health Sciences, University for Development Studies, P.O. Box TL 1883, Tamale, Ghana; Department of Nursing, School of Allied Health Sciences, University for Development Studies, Tamale, Ghana

**Keywords:** Type 2 diabetes, Weight management behaviours, Healthcare professionals, Counselling, Ghana

## Abstract

**Background:**

Weight loss has been shown to influence the health outcomes of type 2 diabetes patients. Providing weight management counselling to diabetes patients may help them adopt appropriate weight management behaviours to lose weight. This study determined the association between self-reported receipt of healthcare professional’s weight management counselling and the weight management behaviours of type 2 diabetes patients.

**Methods:**

This cross-sectional study was conducted among 378 type 2 diabetes mellitus patients seeking care from two hospitals. Using a questionnaire, participants’ weight management behaviours were assessed as well as receipt of healthcare professional’s weight management counselling.

**Results:**

Half (51.3 %) of the participants reported receipt of healthcare professional’s weight management counselling in the last 12 months. Half of the participants ever tried to lose weight. Fewer than half of the participants reported modifying their dietary habits (45.5 %) or engaging in exercise (48.7 %) to lose weight. Those who reported receipt of weight management counselling were more likely to report ever trying to lose weight (AOR 43.0, 95 % CI 23.0–81.6; *p* < 0.001), modifying their dietary habits (AOR 22.5, 95 % CI 13.0–39.19; *p* < 0.001), and engaging in exercise (AOR 13.0, 95 % CI 7.8–21.7; *p* < 0.001) to lose weight.

**Conclusion:**

Participants engaged in varied weight management behaviours. Receipt of health care professionals’ weight management counselling was associated to participants’ reported participation in weight management behaviours. Weight management counselling from health care professionals may support the adoption of weight management behaviours in type 2 diabetes mellitus patients.

## Background

Diabetes is a global public health concern. About 382 million adults aged 20–70 years are currently living with diabetes and projected to rise to 592 million people by the year 2035 (Guariguata et al. 2014). According to the WHO, 10 % of adults aged 25 and above had raised blood glucose in 2013 (WHO 2013). Every 6 s, a person dies of diabetes, making it responsible for 5.2 million deaths in 2013 (Guariguata et al. 2014; WHO 2013). Type 2 diabetes is the most frequent, responsible for 90–95 % of all diabetes cases (Tuei et al. 2010).

About 80 % of the people with diabetes live in low-and middle-income countries, affecting 19.8 million adults in Sub-Saharan African countries (Guariguata et al. 2014). The absence of extensive studies on diabetes in Ghana makes it difficult to quote the current prevalence of the disease in the country. However, the few ones available estimate that about 6 % of adult Ghanaians are living with diabetes (Danquah et al. 2012).

The rising prevalence of overweight and obesity in Ghana and globally has been linked to the increase in the prevalence of diabetes (Mogre et al. 2012; 2014a, b; Gregg et al. 2009; Mokdad et al. 2003; WHO 2011). Overweight and obesity are associated with an increased risk of developing type 2 diabetes (WHO 2013; Chan et al. 1994). Type 2 diabetes patients’ populations are more likely to have higher prevalence of excess weight compared to the general population (Danquah et al. 2012; Daousi et al. 2006). About 50–90 % type 2 diabetes patients in Ghana (Daousi et al. 2006), Nigeria (Fadupin et al. 2004), India (Kamath et al. 2011) and the US (McTigue et al. 2006) are either overweight or obese. Excess body weight in persons living with diabetes is linked to increased risk of death from all causes, cardiovascular diseases and some forms of cancer (Calle et al. 2003). Every 5 kg/m^2^ higher body mass index beyond 25 kg/m^2^ was associated with about 30 % higher overall mortality as reported by the Prospective Studies Collaboration (2009). Conversely, a reduction in body weight lowers the risk of developing type 2 diabetes (Shulman 2005; Sullivan et al. 2005).

Several clinical trials have demonstrated the significant impact of weight loss on reducing the risk of developing diabetes and control of diabetes to risk of diabetes complications (Look AHEAD Research Group 2006; Diabetes Prevention Program Research Group 2000). In fact one study estimates that for every two pounds of weight loss, there is a potential 16 % reduction in risk of developing diabetes (Hamman et al. 2006). A modest weight loss ≥5 but <10 % of one’s initial weight at 1 year was found to be associated with significant improvements in cardiovascular risk factors such as glycaemia, blood pressure, triglycerides, and HDL cholesterol in the Action for Health in Diabetes (Look AHEAD) study (Wing et al. 2011). Other reported benefits of weight loss in type 2 diabetes mellitus include positive changes in quality of life, mobility and physical and sexual function (Wilding 2014). Thus weight management is an important aspect of diabetes self-care management. Diabetes self-management involves a modification in lifestyle behaviours such as eating a healthy diet, participating in regular physical activity, attaining and maintaining a healthy body weight, reducing alcohol intake and quitting/avoiding smoking (Agborsangaya et al. 2013; Cheng and Barnes 2013; Prevention I, TYPE DO 2011).

Central and very critical to diabetes self-management care is the health care professional. A collaborative relationship between the health care professional and the diabetes patient is encouraged. In this collaborative relationship the health care professional is encouraged to provide the diabetes patient with support and guidance to help her/him manage her/his condition effectively (Linda 2008). Health care professionals can provide support by counselling their diabetes patients on weight management and encouraging them to adopt weight management behaviours. One study found that healthcare professionals’ advice on diabetes risk influenced the adoption of healthy lifestyles (Okosun et al. 2012). Two studies among diabetes patients found that participants who reported receipt of healthcare professional’s advice for specific diabetes self-management behaviours were more likely to report following the corresponding behaviour (Vaccaro et al. 2012; Dorsey and Songer 2011). Notwithstanding the positive effect of healthcare professionals’ advice or counselling on the adoption of healthy lifestyles including weight management behaviours as well as several organizations recommending physician screening and counselling for overweight and obesity, most healthcare professionals do not counsel their patients to adopt healthy lifestyles including weight management behaviours (Brotons et al. 2005; Force 2003). In the US, a little lower than 50 % of obese patients reported receipt of weight management advice from health care professionals (Galuska et al. 1999; Ko et al. 2008).

A number of challenges have been reported by health care professionals regarding the provision of weight management counselling. These challenges include the unavailability of tools, inadequate training, low reimbursement, inadequate staffing and lack of time, and lack of confidence in their own abilities and in the effectiveness of weight management measures in general (Temple 1999; Visser et al. 2008).

Studies on weight management behaviours and the influence of health care professionals’ weight management counselling on the adoption of weight management behaviours are non-existent in Sub-Saharan Africa including Ghana. This study evaluated the weight management behaviours followed by type 2 diabetes patients. The study further assessed the proportion of type 2 diabetes patients who reported receipt of weight management counselling from health care professionals. Finally, we determined the associations between self-reported receipt of healthcare professional’s weight management counselling and self-reported adoption of weight management behaviours among the type 2 diabetes patients.

## Methods

### Participants

Data for this study came from the Tamale Diabetes Study (TDS). The TDS is a small, authors’ funded, unregistered cross-sectional study conducted among previously diagnosed type 2 diabetes mellitus patients seeking care from two hospitals (Tamale Teaching Hospital and Tamale Central Hospital) in the Tamale Metropolis, Ghana. Located about 500-600 km North of Accra, the Capital city of Ghana, Tamale is the second largest city by area size in Ghana with a population of 562, 919 inhabitants based on the 2012 Ghana Population and Housing Census. The TDS collects varied health information of the diabetes patients including clinical measurements, anthropometric data, demographics, and weight management behaviours, self-assessment of weight status and receipt of weight management counselling as well knowledge on the treatment and management of the condition. For the purposes of the current study, participants’ data on demographics, weight management behaviours and receipt of weight management counselling were included into the analysis.

**Fig. 1:**
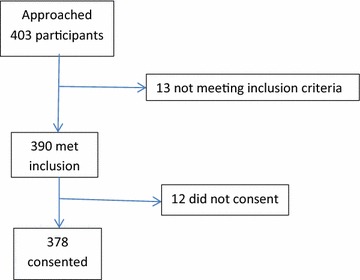
Selection of participants

From April–July 2014, 403 participants were approached; 390 met the inclusion and exclusion criteria; however 378 participants (response rate = 96.9 %) agreed to participate and were included into the study (shown in Fig. 1). Reasons for non-participation were generally personal. Participation in the study was voluntary and all participants provided informed consent. The study was approved and granted permission by the Research Unit of the Tamale Teaching Hospital.

### Inclusion criteria

Those self-reporting health care professional diagnosis of type 2 diabetes; did not report a type but reported being diagnosed after the age of 30 years and/or reported duration of diagnosis ≥1 year were eligible to participate in the study.

### Exclusion criteria

All pregnant women who reported having diabetes were excluded due to the different recommendations for weight management behaviours in this group. Those self-reporting a history of type 1 DM, heart failure, myocardial infarction, acromegaly, hypogonadism and/or any other chronic diseases were also excluded. Furthermore, those who were on active drug treatment for weight loss and had been diagnosed with the condition for less than a year were not eligible to participate.

### Measures

Participants reported their engagement in weight management behaviours, including whether they ever tried doing any of the following (yes/no) as a result of being diagnosed with diabetes; tried to lose or gain weight or did neither of these. If participants reported ever trying to lose weight, they were asked to indicate the behaviours they adopted (i.e. modified dietary habits, engaged in physical activity/exercise or used drugs). Reported modification of dietary habits to lose weight included changing the type of food, the energy content of the food (i.e. high calorie diet and low-calorie diets) and the frequency of meals (i.e. eating regularly or skipping meals). Quantities of foods consumed were not assessed due to lack of uniform serving sizes in Ghana. Participants engagement in physical activities to lose weight were assessed by asking participants to indicate the number of times per week (i.e. once, twice and ≥3) and time spent (i.e. <30, 30–60 and >60 min) doing activities such as walking, bicycling, jogging, skipping etc. Participants were asked whether they had weight management and physical activity goals i.e. how much weight they intended to lose or the frequency and duration of exercise they intended to engage in for the next few months. However, these data were inconsistent and had several missing values and as result were excluded from the analysis. The frequency with which participants measured their weight status was also assessed (i.e. 0 times per week, once, 2–5 times, 6–15 times and 16 or more times).

To assess receipt of healthcare professional’s weight management counselling, participants were asked the question “Have you ever been counselled on weight management (i.e. to lose/control your weight; engage in exercise, modify your diet) by a health care professional (physician, nurse, diabetes educator, dietician/nutritionist, pharmacists, biomedical scientist, among others) in the last 12 months (yes/no)”. Participants who answered “Yes” to this question were considered to have received weight management counselling from a health care professional and noted as counselled; those who responded “No” were considered to have not received weight management counselling and noted as not counselled. Participants were considered to have received weight management counselling if they responded yes to any of the following: being spoken to about one’s weight; diet and/or exercise habits. All these were measured using paper questionnaires that were administered by one of the authors to the participants. The questionnaires were administered by the author in a face-to-face setting for those who could neither speak nor write in the English Language and self-completed by those who could speak and write in the English language. All these were done in a secluded area. The items of the questionnaire were adapted from previously published articles (Agborsangaya et al. 2013; Ekezue and Platonova 2012; Grandy et al. 2012). The questionnaire showed a good level of internal consistency (Cronbach alpha = 0.620).

### Covariates

Socio-demographic variables such as gender, age, marital status, religious following, educational status and number of years since diabetes diagnosis were also collected using the questionnaire. These socio-demographic variables were categorized as follows: age (<50 and ≥50 years), educational status (no education, low and high level of education), marital status (married and not married) and number of years since diabetes diagnosis (<5 and ≥5 years). Low level of education included those who reported to have attained basic level of education (Primary and Junior high). High level of education included those who reported to have attained senior high level of education or higher. All participants who reported being single or divorced was considered as not married.

### Anthropometric and clinical measurements

Participants’ weight and height were measured with standard protocols and computed into body mass index (BMI: weight in kilograms divided by the square of height in metres). In accordance with the World Health Organisation’s BMI classifications, participants with a BMI ≤ 18.5 kg/m^2^ was considered underweight, 18.5–24.9 kg/m^2^: normal weight, 25.0–29.9: 25–29.9 kg/m^2^: overweight, and BMI ≥ 30 kg/m^2^: obese (WHO 2000).

Participants’ blood pressure (BP) was measured following standard procedures. Using a standard mercury sphygmomanometer, BP was measured while the participant was seated with her/his arm resting at the level of the heart. Systolic blood pressure (SBP) and diastolic blood pressure (DBP) was recorded to the nearest 2 mmHg. A second measurement was done for a participant if the first measurement was found to be elevated for confirmatory purposes. Elevated blood pressure denoted a mean BP ≥ 140/90 mmHg and/or documented anti-hypertensive treatment (Chalmers et al. 1999).

### Statistical analysis

All data analyses were conducted using IBM SPSS Statistics 21. Frequency distributions were used to describe all categorical variables including socio demographic variables and weight management behaviours. Weight management behaviours of the participants were stratified by receipt of weight management counselling from a healthcare professional and compared using Fisher’s exact test.

Controlling for confounders such as age, gender, educational status, diabetes duration, weight, and blood pressure status, multivariable logistic regression analysis using forward selection (conditional) method was carried out to examine the association between reported receipt of health care professionals’ weight management counselling and reported participation in weight management behaviours. In each of the logistic regression models, receipt of health care professionals’ weight management counselling was considered as the independent variable and the weight management behaviours engaged by the participants considered as the dependent variables (ever tried to lose weight, modified dietary habits, engaged in physical activity, and has a weight management plan). Four logistic regression models were computed corresponding to the four dependent variables. The results were presented as Adjusted Odds Ratios (AOR) at 95 % Confidence Intervals. Having a physical activity plan was not included into the analysis due to the fact that the univariate analysis did not yield any statistical significant associations for it.

The frequencies reporting trying to gain weight and using drugs to lose weight were small making them statistically unreliable and as a result were excluded from the logistic regression models. In all statistical analyses, a *p* value of <0.05 was considered significant at 95 % confidence interval.

## Results

### General characteristics of the participants

Table 1 displays the general characteristics of the participants stratified by receipt of healthcare professional’s weight management counselling. Participants were frequently female (61.5 %, n = 246), younger than 50 years of age (59.3 %, n = 224) and less than half (45.6 %, n = 172) had been living with the condition ≥5 years (shown in Table 1). About 56 % of the participants had high level of education; 58.7 % were hypertensive and 58.7 % were either overweight/obese. Participants with elevated blood pressure were more likely than their normotensive counterparts to report receipt of healthcare professional’s weight management counselling. Receipt of healthcare professional’s weight management counselling did not differ significantly by gender, weight status, age and duration of diabetes.

**Table 1:** General characteristics of the participants stratified by receipt of healthcare professional weight management counselling

Variable	Total (n = 378)	Receipt of healthcare professional weight management counselling		
		Yes (n = 194)	No (n = 184)	*p* value
Gender				
Male	132 (34.9 %)	62 (32.0 %)	70 (38.0 %)	0.236
Female	246 (65.1 %)	132 (68.0 %)	114 (62.0 %)	
Mean BMI (Kg/m^2^)	26.77 ± 5.66	27.98 ± 6.20	25.50 ± 4.72	<0.001
Weight status				
Overweight/obese	222 (58.7 %)	134 (69.1 %)	88 (47.8 %)	0.549
Normal weight	156 (41.3 %)	60 (30.9 %)	96 (52.2 %)	
Elevated blood pressure				
Yes	256 (67.7 %)	144 (74.2 %)	112 (60.9 %)	0.006
No	122 (32.3 %)	50 (25.8 %)	72 (39.1 %)	
Age				
≥50	154 (40.7 %)	88 (45.4 %)	66 (35.9 %)	0.075
<50	224 (59.3 %)	106 (54.6 %)	118 (64.1)	
Duration of diabetes				
<5 years	206 (54.5 %)	104 (53.6 %)	102 (55.4 %)	0.757
≥5 years	172 (45.5 %)	90 (46.4 %)	82 (44.6 %)	

### Prevalence of weight management behaviours compared with the receipt of healthcare professional’s weight management counselling

With a preponderance to women (57.7 vs. 45.5 %, *p* = 0.024), 59.3 % of the participants reported ever trying to lose weight in the last 12 months; were more likely to have low level of education (56.2 %, n = 118 vs. 50.0 %, n = 84; *p* = 0.006) and married (50.7 %, n = 150 vs. 63.4 %, n = 52; *p* = 0.046). However, participants reporting ever trying to lose weight did not differ significantly by duration of diabetes diagnosis (57.0 %, n = 98 vs. 50.5 %, n = 104; *p* = 0.208) and age (50.0 %, n = 112 vs. 58.4 %, n = 90; *p* = 0.116).

Weight management behaviours adopted by the participants to lose weight included modification of dietary habits (76.8 %); engaging in exercise (82.1 %) and use of drugs (4.5 %). Significantly (*p* = 0.003) more women (51.2 %, n = 126) than men (34.8 %, n = 46) modified their dietary habits to lose weight. No significant gender differences were noted in participants who participated in exercise/physical activity (52.0 vs. 42.4 %, *p* = 0.084) to lose weight and had a weight management plan (21.1 vs. 19.7 %, *p* = 0.791). Participants reporting participation in exercise/physical activity and using drugs to lose weight did not differ significantly by age, duration of diabetes and marital status. However, participants who had low educational level (57.1 %, n = 120) were more likely (*p* = 0.021) than those having high (45.0 %, n = 72) educational levels to modify their dietary habits to lose weight. Furthermore, those reporting participation in exercise/physical activity differed significantly by level of education (47.6 %, n = 100 vs. 56.0 %, n = 94; *p* = 0.121).

A little over half (51.3 %) of the participants reported receipt of healthcare professional’s weight management counselling in the last 12 months. Reported Receipt of healthcare professional’s weight management counselling did not differ significantly by gender (females: 53.4 %, n = 132; males: 47 %, n = 62); age (47.3 %, n = 106 vs. 57.1 %, n = 88; *p* = 0.075); duration of diabetes diagnosis (52.3 %, n = 90 vs. 53.6 %, n = 104; *p* = 0.757); marital status (50.0 %, n = 148 vs. 56.1 %, n = 46; *p* = 0.382) and level of education (48.6 %, n = 102 vs. 54.8 %, n = 92; *p* = 0.535).

The reported weight management behaviours of the participants were stratified according to reported receipt of healthcare professional’s weight management counselling as presented in Table 2. A higher proportion of participants who reported receipt of healthcare professional’s weight management counselling in the last 12 months; reported ever trying (89.7 vs. 27.2 %, *p* < 0.001) to lose weight and engaged in diet modification to lose weight (85.1 vs. 48.0 %, *p* = 0.003) compared to those who reported not receiving healthcare professional’s weight management counselling.

**Table 2:** Proportion of adults aged 20 years and older with type 2 diabetes (n = 378) engaging in weight management behaviours stratified by reported receipt of weight management counselling in the last 12 months

Variable	Total (n = 378)	Receipt of healthcare professional counselling		
		Yes (n = 194)	No (n = 184)	*P* value
Weight management behaviours				
Ever tried to lose weight	224 (59.3 %)	174 (89.7 %)	50 (27.2 %)	<0.001
Ever tried to gain weight	6 (1.6 %)	4 (2.1 %)	2 (1.1 %)	0.686
Neither tried to lose weight nor to gain weight	148 (39.2 %)	16 (8.2 %)	132 (71.7 %)	<0.001
Participated in the following to lose weight	n = 224	n = 174	n = 50	
Modified dietary habits	172 (76.8 %)	148 (85.1 %)	24 (48.0 %)	0.003
Engaged in exercise	184 (82.1 %)	146 (83.9 %)	38 (76.0 %)	0.212
Used drugs	10 (4.5 %)	10 (5.7 %)	0 (0.0 %)	0.068
Has a weight management plan/goal				
Yes	78 (20.6 %)	50 (25.8 %)	28 (15.2 %)	0.015
Has a physical activity plan				
Yes	162 (42.9 %)	82 (42.3 %)	80 (43.5 %)	0.836
Modified dietary habits by eating	n = 172	n = 148	n = 24	
Low-calorie diet	154 (89.5 %)	132 (81.2 %)	22 (91.7 %)	1.000
Skipping of meals	18 (10.5 %)	16 (10.8 %)	2 (4.0 %)	
Frequency of exercise per week	n = 184	N = 146	N = 38	
Once	12 (6.5 %)	10 (6.8 %)	2 (5.3 %)	1.000
2 times	70 (38.0 %)	50 (34.2 %)	20 (52.6 %)	0.041
≥3 times	102 (55.4 %)	86 (58.9 %)	16 (42.1 %)	0.070

Twenty-one percent of the participants had a weight management plan and 42.9 % had a physical activity plan. Those having a weight management plan did not differ significantly by gender (80.3 %, n = 106 vs. 64.7 %, n = 194; *p* = 0.791); age (80.4 %, n = 180 vs. 77.9 %, n = 120; *p* = 0.606); duration of diabetes (75.6 %, n = 130 vs. 82.5 %, n = 170; *p* = 0.099); educational level (76.2 %, n = 160 vs. 83.3 %, n = 140; *p* = 0.097) marital status (79.7 %, n = 236 vs. 78.0 %, n = 64; *p* = 0.759). Having a physical activity plan did not also differ significantly by gender (39.4 %, n = 52 vs. 44.7 %, n = 110; *p* = 0.329); age (40.2 %, n = 90 vs. 46.8 %, n = 72; *p* = 0.207); duration of diabetes (40.7 %, n = 70 vs. 44.7 %, n = 92; *p* = 0.466); educational level (43.8 %, n = 92 vs. 41.7 %, n = 70; *p* = 0.754) and marital status (44.6 %, n = 132 vs. 36.6 %, n = 30; *p* = 0.209).

Multivariable analysis of factors associated with the reported weight management behaviours of the participants are presented in Table 3. Participants who reported receipt of healthcare professional’s weight management counselling were more likely than their counterparts to have ever tried (AOR 43.0, 95 % CI 23.00–81.61; *p* ≤ 0.001) to lose weight; modified their dietary habits (AOR 22.5, 95 % CI 13.00–39.19; *p* ≤ 0.001); and engaged in exercise (AOR 13.0, 95 % CI 7.82–21.65; *p* ≤ 0.001). Participants having low level of education (Junior high school and below) were more likely than those having high level of education to report ever trying to lose weight (AOR 2.6, 95 % CI 1.1–6.3; *p* = 0.007) and participated in exercise/physical activity (AOR 1.9, 95 % CI 1.17–3.22; *p* = 0.010). Participants with elevated blood pressure were more likely to report modifying their dietary habits to lose weight compared to their counterparts with normal blood pressure (AOR 2.7, 95 % CI 1.46–4.81; *p* = 0.001).

**Table 3:** Multivariate associations between demographic factors, receipt of weight management counselling and engaging in some weight management behaviours among Ghanaian adults aged 20 years and older with type 2 diabetes (n = 378)

Variable	B	AOR (95 % Cl)	*p* value	Nagelkerke R square
Tried to lose weight				0.58
Low educational status	0.86	2.4 (1.26–4.42)	0.007	
Received weight management counselling	3.76	43.0 (23.00–81.61)	<0.001	
Modified diet to lose weight				0.52
Female	0.84	2.2 (1.23–3.83)	0.004	
Received weight management counselling	3.11	22.5 (13.00–39.19)	<0.001	
Elevated blood pressure	0.98	2.7 (1.46–4.81)	0.001	
Participated in exercise to lose weight				0.41
Low educational status	0.67	1.9 (1.17–3.22)	0.010	
Received weight management counselling	2.57	13.0 (7.82–21.65)	<0.001	
Overweight/obese	0.88	2.4 (1.45–3.98)	0.001	
Has a weight management plan				0.11
Low educational status	0.50	1.6 (0.97–2.78)	0.043	
Overweight/obese	1.46	4.3 (2.29–8.04)	<0.001	

Factors entered into the logistic regression blocks were receipt of weight management counselling; gender, age, educational level, marital status, duration of diabetes after diagnosis. Forward Selection (conditional) stepwise method was employed for all regression analysis

*AOR* adjusted odds ratio

## Discussion

Given the risk of diabetes complications associated with excess weight in type 2 diabetes mellitus, we assessed the weight management behaviours of Ghanaian adults’ aged 20 years and older living with type 2 diabetes mellitus. The study further assessed the association between reported receipt of healthcare professional’s weight management counselling with reported participation in weight management behaviours of the participants. The weight management behaviours reported by the participants were congruent with evidence-based recommendations for weight management including modification of dietary habits and participation in exercise/physical activity. Other methods of losing weight such as the use of drugs were less frequent. A little over half (51.3 %) of the participants received healthcare professional’s weight management counselling in the last 12 months. Receipt of weight management counselling was associated with methods adopted to lose weight including ever trying to lose weight, modifying dietary habits, participating in exercise/physical activity and having a weight management plan.

The reported estimates of weight loss attempts in this study can be said to be among the lowest reported in the literature. Using data from the 2006 National Health Interview Survey, an in-person survey of the US non-institutionalized population, reported weight loss attempts of approximately 75 % of diabetic overweight and obese patients (Dorsey and Songer 2011). Based on data from the Behavioural Risk Factor Surveillance System (BRFSS), one study reported a prevalence of weight loss attempts of 60–72 % for people with diabetes in the US (Zhao et al. 2009; Bish et al. 2005). However, our findings are consistent with the findings of Agborsangaya et al. Using data from the 2011 Survey on Living with Chronic Diseases in the Canada Diabetes Component (SLCDC-DM), Agborsangaya et al. (2013) reported a weight loss attempt estimate of approximately 56 % among Canadian adults aged 20 years and older living with type 2 diabetes. The relatively low prevalence of weight loss attempts in this study could be attributed to the fact that a relatively low proportion of them received healthcare professional’s weight management counselling. It could also be due to the acceptance of excess body weight as a sign of well-being and beauty in the general population in Ghana and other sub-Saharan African countries (Addo et al. 2009). Findings from previous study indicate both diabetes patients and the general population underestimate their weight status (Mogre et al. 2013, 2014c, 2015).

The reported prevalence of 51.3 % of the participants receiving healthcare professional’s weight management counselling is lower than estimates reported from previous studies conducted elsewhere. Studies conducted in the United States and the UK have reported estimates ranging from 58 to 89 % among diabetes patients (Dorsey and Songer 2011; Galuska et al. 1999; Ko et al. 2008; Ekezue and Platonova 2012; Jackson et al. 2005, 2013; Simkin-Silverman et al. 2005). The relatively low prevalence of reported weight management behaviours and healthcare professional’s weight management counselling among the diabetes patients probably suggest that patients might not be getting the needed quality of care expected for their condition. Evidence from elsewhere have identified barriers to healthcare professional’s provision of weight management counselling to include time constraints, lack of adequate knowledge and skills to provide weight management counselling, inadequate training and confidence as well as cultural and contextual factors that accept excess body weight (Hiddink et al. 1995; Harris et al. 1999; Ruelaz et al. 2007; Kenner et al. 1999; Leverence et al. 2007; Foster et al. 2003; Michie 2007). Furthermore, the low prevalence of receipt of healthcare professional’s weight management counselling could also be due to the less developed and weak healthcare systems for the management of chronic diseases in the study setting including inadequate health care personal, poor patient compliance, and unavailability of in-service training programmes to build the capacity of healthcare professionals (Mbanya et al. 2006, 2010). Future studies should elaborately explore these barriers and the influence on diabetes care.

After controlling for age, gender, educational status, duration of diabetes, and marital status as well as body weight and blood pressure, self-reported receipt of healthcare professional’s weight management counselling was significantly associated with reported participation in a number of weight management behaviours. Recognizing our inability to determine if health care professional counselling preceded behaviour, our findings nonetheless are consistent with several studies reporting a positive association between reported receipt of health care professional’s advice and reported engagement in weight management behaviours (Agborsangaya et al. 2013; Jackson et al. 2013; Otero-Sabogal et al. 2010; Siddiqui et al. 2010; Rose et al. 2012; Vaccaro et al. 2012). The findings of our study presents additional evidence on the importance of healthcare provider’s support either in the form of providing information or participating in decision making to help modify and improve self-management behaviours of diabetes patients including weight management. In a systematic review, Rose et al. (2012) concluded that physician advice could have a positive effect on patients’ participation in weight loss behaviours. Their meta-analysis showed that healthcare provider’s weight loss advice had a significant impact on patients’ attempts to lose weight. Just a brief counselling encounter with a patient may play a significant role in his/her weight management behaviour (Rose et al. 2012). This suggest that improving the quality of healthcare provider’s communication may lead to better self-care management, resulting in improved health outcomes including maintaining healthy weight (Heisler et al. 2002, 2007, 2009). Although, health care professional advice is significant, successful weight loss and weight control and sustained weight management behaviours are influenced by several factors including behavioural interventions, patient education, family and social support (Dorsey and Songer 2011).

Gender may be important in the adoption of weight management behaviours in diabetes patients as more women than men reported modifying their dietary habits to lose weight. This is consistent with several studies conducted among diabetes patients (Agborsangaya et al. 2013; Dorsey and Songer 2011; Zhao et al. 2009). Healthcare providers should take note of these gender differences in providing weight management counselling to patients.

Another important finding of this study was that level of education was found to be associated to ever trying to lose weight and engaging in exercise to lose weight. Interestingly, those having low level of education were more likely than those having high level of education to report ever trying to lose weight and participating in exercise to lose weight. Contrary findings have been reported in the general population in which the perception of trying to lose weight increased with increasing level of education (Bish et al. 2005; Kruger et al. 2004; Serdula et al. 1999). Inconsistent associations between level of education and perception of trying to lose weight and adopting weight management behaviours have been reported in diabetes populations. While some studies have reported the perception of trying to lose weight and participation in weight management behaviours, to increase with increasing educational levels (Agborsangaya et al. 2013; Zhao et al. 2009), others have either reported findings similar to ours (Gurka et al. 2006) or have found no association at all (Dorsey and Songer 2011).

Although there was no significant association between receipt of weight management counselling and weight status, overweight/obese participants were more likely than their normal weight counterparts to report participation in weight management behaviours such as exercising to lose weight and having a weight management plan. Plausibly, these participants adopted weight management behaviours because they felt it was a healthy lifestyle although there were not advised to do so.

### Strengths and limitations

To the best of our knowledge this is the first study that assessed the association between reported receipt of healthcare professional’s weight management counselling and reported engagement in weight management behaviours among type 2 diabetes patients in Sub-Saharan Africa. It thus provides baseline data for further studies in the future. One other strength of this study is that it assessed reported weight management behaviours of type 2 diabetes patients irrespective of their weight status. This brings to bear that type 2 diabetes patients may be engaging in weight management behaviours irrespective of their weight status.

It is important to note the limitations of this study. All data were self-reported, thus subject to recall and social desirability biases. This is a cross-sectional study making it impossible to establish cause and effect. Health care professionals’ weight management counselling was reported by the study participants and not by their health care provider. As such participants may have reported not receiving weight management counselling when in fact they had received it. This sample is based on those seeking health care from two health institutions, therefore it cannot be generalized to those receiving care from other health institutions as well as those having the condition and not seeking care at all or seeking care from herbal centres. Other factors may influence our findings that we were unable to assess. For instance we were not able to assess contextual factors such as social network, family, friends and socio-economic status that may help to explain the adoption or non-adoption of weight management behaviours (Simkin-Silverman et al. 2005). Data on socio-economic status that have been shown to influence the adoption of weight management behaviours were not collected (Agborsangaya et al. 2013). Participants were unwilling to provide information on their socio-economic status. In addition, some factors that have been shown to be associated with non-receipt of health professional weight management counselling including poor access to health care facilities and healthcare provider-patient relationship were not measured (Honda 2004; Sinclair et al. 2008). Furthermore, we were unable to measure the nature and frequency of weight management counselling participants received from health care professionals or participants’ exposure to other strategies of weight management strategies. It’s worth investigating in future the type and form of weight management counselling provided to patients by healthcare professionals. We did not collect information on how recent participants received weight counselling from health care professional prior to the study. The timing of weight management counselling and receipt of counselling are important determinants of adopting weight management behaviours that needs to be explored further. Finally our study did not assess whether participation in weight behaviours will be associated with reduction in weight or improvement in the disease status of the participants. This makes it difficult to determine the effectiveness of counselling in weight management in type 2 diabetes. Future studies should explore this phenomenon.

### Implications for practice

The findings of this study add to the literature that weight management counselling provided by health care professionals’ may influence the adoption of weight management behaviours by type 2 diabetes mellitus patients. Our findings that, participants who reported receipt of healthcare professional’s weight management counselling were more likely to report participation in weight management behaviours suggests that advice from a healthcare professionals may help bridge the behaviour-intention gap (Jackson et al. 2013). Together with other studies, our results provide support for the recommendation that health care professionals including physicians, diabetes educators, nurses, dieticians, among others, should discuss weight management with type 2 diabetes mellitus patients (Agborsangaya et al. 2013; Dorsey and Songer 2011; Ekezue and Platonova 2012).

## Conclusion

A relatively low proportion of these Ghanaian adult type 2 diabetes patients reported participation in weight management behaviours including modification of dietary habits and engagement in exercise. A relatively low proportion of them reported receipt of healthcare professional’s weight management counselling. Reported receipt of healthcare professional’s weight management counselling was associated with reported weight management behaviours of these type 2 diabetes mellitus patients. It suggests healthcare professional advice may be important in addressing the issue of weight management in type 2 diabetes patients. Future research should aim to identify factors that might affect health care professional’s provision of weight management counselling to diabetes patients in the study setting.
